# Investigaciones cualitativas en salud pública publicadas en revistas biomédicas colombianas entre el 2011 y el 2021

**DOI:** 10.7705/biomedica.6476

**Published:** 2023-03-30

**Authors:** Rodolfo Rodríguez-Gómez

**Affiliations:** 1 Asesor metodológico en investigación en salud, Bogotá, D.C., Colombia Bogotá, D.C., Colombia

**Keywords:** investigación cualitativa, salud pública, artículo de revista, revistas electrónicas, entrevista, Colombia, Qualitative research, public health, journal article, electronic journals, interview, Colombia

## Abstract

**Introducción.:**

La investigación cualitativa busca comprender el sentido y la perspectiva de los individuos e indaga teniendo en cuenta el contexto. Este paradigma permite la interpretación y el estudio de los fenómenos desde su propia complejidad. En salud pública, la investigación cualitativa ha ganado terreno, pues permite estudiar aspectos subjetivos del proceso salud-enfermedad.

**Objetivo.:**

Describir y analizar las investigaciones cualitativas en temas de salud pública publicadas en revistas colombianas entre el 2011 y el 2021.

**Materiales y métodos.:**

Se llevó a cabo un estudio descriptivo sobre las investigaciones cualitativas en salud pública publicadas en revistas colombianas entre el 2011 y el 2021.

**Resultados.:**

Se incluyeron 81 artículos. La revista con mayor cantidad de publicaciones fue la *Revista de Salud Pública* (44,4 %). El año con mayor producción correspondió al 2019 y el principal diseño fue la teoría fundamentada (17,3 %). En el 79 % de los artículos, las mujeres fueron el autor principal y la enfermería constituyó la profesión más frecuente de formación de pregrado. El tema más frecuente fue el VIH/sida (12,3 %), seguido por el cáncer (11,1%). En el 24,6 % de los estudios, se utilizó un *software* para el análisis.

**Conclusiones.:**

Las investigaciones cualitativas en salud pública han tenido una dinámica variable en la producción científica entre el 2011 y el 2021. Pese a sus bondades, la adopción de programas informáticos para el análisis cualitativo es escasa. La enfermería se destaca como el área que concentra la mayor cantidad de estudios cualitativos en salud pública con aportes en temáticas como COVID-19 y VIH/sida.

## Abstract (en)

**Introduction:**

Qualitative research seeks to understand the meaning and perspective of individuals and investigates considering the context. This paradigm allows the interpretation and study of phenomena from their own complexity. In public health, qualitative research has gained ground, as it allows the study of subjective aspects of the health-disease process.

**Objective:**

To describe and analyze qualitative research on health issues published in Colombian journals between 2011 and 2021.

**Materials and methods:**

A descriptive study on qualitative health research published in Colombian journals between 2011 and 2021 was carried out.

**Results:**

Eighty-one articles were included. The journal with the highest number of publications was the *Revista de Salud Pública* (44.4%). The year with the highest production corresponded to 2019 and the main design was the fundamental theory (17.3%). In 79% of the articles, women were the main author and nursing was the most frequent profession of undergraduate training. The most frequent topic was HIV/AIDS (12.3%), followed by cancer (11.1%). In 24.6% of the studies, a software was used for the analysis.

**Conclusions:**

Qualitative research in public health has had a variable dynamic in scientific production between 2011 and 2021. Despite its benefits, the adoption of computer programs for qualitative analysis is rare. Nursing stands out as the area that concentrates the largest number of qualitative studies in public health with contributions on topics such as COVID-19 and HIV/AIDS.

La investigación cualitativa es aquella que, enmarcada en el paradigma naturalista, busca comprender los significados, el sentido y la perspectiva de los individuos y, al mismo tiempo, cómo esas perspectivas se construyen según los contextos sociales y culturales ^1^. Es un tipo de investigación flexible que permite una interacción cíclica entre los datos y el investigador, siempre con la pregunta de investigación como elemento guía del proceso.

El paradigma cualitativo se fundamenta en el razonamiento inductivo, usa múltiples fuentes de datos como textos e imágenes y busca entender la particularidad ^1^, al tiempo que estudia los fenómenos desde su propia complejidad ^2^. Dado que busca obtener datos en su entorno natural, a la investigación cualitativa también se le denomina *naturalista*. Comprende investigaciones que generan resultados a los que no se llega por medio de análisis estadísticos ^3^ y puede dar cuenta de las experiencias personales, las motivaciones, los comportamientos y los movimientos sociales ^3^.

La investigación cualitativa no es producto de una sola rama del saber, ni resultado de la evolución lineal del conocimiento. Por el contrario, su nacimiento y desarrollo han sido intrincados, y en ello han contribuido en grado variable la filosofía, la antropología, la sociología, la psicología y la educación. Esto es relevante, pues varias de estas áreas del saber también aportan en el complejo campo de la salud pública, en el cual, a su vez, participan la demografía, la economía de la salud, la epidemiología y la medicina clínica, entre otras.

La investigación cualitativa guarda distancia con la medición y la cuantificación, para dar cabida a la comprensión, la interpretación y el surgimiento de la teoría, como es el caso de la teoría fundamentada ^3^. Las herramientas cualitativas son diversas como, por ejemplo, los grupos focales, los diarios de campo, la observación participante, los relatos de vida y, principalmente, las entrevistas. Cabe resaltar que, en este tipo de investigación, el investigador representa el principal instrumento; por ende, debe contar con habilidades como pensar de manera crítica, interpretar y analizar en contexto ^4^.

Durante mucho tiempo existió una profunda brecha entre la investigación cualitativa y el área de la salud, donde ha dominado el paradigma cuantitativo. No obstante, en los últimos años, la salud pública ha encontrado en la investigación cualitativa un aliado para dar respuesta a preguntas que no son propias del paradigma cuantitativo y a las que no se puede dar respuesta con él. Como aportes de la investigación cualitativa al área de la salud pública, vale la pena destacar el abordaje de aspectos culturales del proceso de salud-enfermedad, además del estudio sobre creencias, comportamientos, actitudes, motivaciones y percepciones de problemas de salud pública por parte de diferentes actores sociales ^5^.

Asimismo, la investigación cualitativa puede aportar en el análisis de las barreras de los programas de salud pública, en el estudio de aspectos subjetivos como el autocuidado, y en la aproximación a las experiencias con el sistema de salud, las enfermedades, los profesionales de la salud y los tratamientos.

Con las múltiples estrategias para abordar los fenómenos, la investigación cualitativa contribuye a complementar el arsenal investigativo de la salud pública ^6^. Además, en sintonía con la multidisciplinariedad propia de la salud pública, los métodos y enfoques de la investigación cualitativa se ajustan de forma idónea a ella, ya que, ante los problemas emergentes, se han gestado nuevas preguntas que requieren diferentes abordajes y aproximaciones. Los tópicos en los que la investigación cualitativa puede contribuir al área de la salud pública son múltiples como, por ejemplo, salud reproductiva, VIH/sida, salud mental, salud materno-infantil, atención primaria en salud, violencia, salud comunitaria, consumo de sustancias psicoactivas y suicidio, entre otros.

Por tanto, considerando el creciente interés del sector de la salud pública en Colombia por la investigación cualitativa, el propósito de este estudio fue revisar la literatura y hacer un análisis crítico de la información existente sobre investigaciones cualitativas en temas de salud pública, y que fueron publicadas en revistas biomédicas colombianas entre los años 2011 y 2021.

## Materiales y métodos

Se llevó a cabo una revisión de la literatura enfocada en artículos originales producto de investigaciones cualitativas que se encuentran disponibles en internet y que se han publicado en revistas biomédicas colombianas durante el periodo 2011-2021. Este estudio toma indicadores de contenido y metodológicos, pero no incluye indicadores de productividad y citación. Se consultaron revistas indexadas en sistemas de información como Latindex, Scielo, Redalyc y Scimago, entre otros. Se incluyeron artículos originales y se excluyeron artículos con enfoques mixtos (cualitativo y cuantitativo). Se recuperó información que se consideró relevante, como el nombre del artículo, la revista, el año de publicación, el tema principal, el autor principal, el número de autores, el número de participantes, el uso de *software*, el enfoque del estudio, el tipo de muestreo y el número de referencias.

No se realizó cálculo de muestra ni se estableció restricción idiomática. La búsqueda de información se hizo teniendo en cuenta el listado de revistas biomédicas colombianas consultado en Publindex (https://scienti.minciencias.gov.co/publindex/#/noticias/lista). Se consultaron los sitios web de cada una de las revistas biomédicas y se realizaron las búsquedas respectivas. En cada sitio web, se utilizó el filtro para establecer un periodo de búsqueda entre los años 2011 y 2021. Se incluyeron artículos producto de investigaciones cualitativas relacionados con temas de salud pública, publicados en revista colombianas.

### Criterios de selección

1. Artículos cualitativos publicados entre el 2011 y el 2021.
2. Artículos publicados en revistas colombianas del área de la salud.
3. Artículos enfocados en temas de salud pública.

### Criterios de exclusión

1. Estudios mixtos (cualitativos/cuantitativos)

La indexación de las revistas se verificó en Publindex (Sistema de Indexación y Homologación de Revistas Especializadas de Ciencia Tecnología e Innovación - Colombia), y visitando el sitio web de cada revista donde se especifica la indexación en portales como Scimago, Latindex, Scielo y Redalyc.

### Terminología de búsqueda

Los términos utilizados fueron:

Descriptores (DeCS): investigación cualitativa, análisis cualitativo, entrevista, entrevistas grupales, grupos focales y teoría fundamentada.

Términos libres: cualitativo, fenomenológico, fenomenología, etnográfico, hermenéutico, técnica cualitativa y estudio de caso.

Se buscaron artículos en 47 revistas colombianas del área de la salud. Las búsquedas se llevaron a cabo visitando el sitio de web de cada revista y utilizando las palabras clave en los buscadores de cada revista. Las búsquedas se hicieron entre el 1° de septiembre y el 30 de noviembre de 2021 (cuadro 1).

**Cuadro 1:** Listado de revistas consultadas

Acta Colombiana de Psicología
Acta Médica Colombiana
Acta Neurológica Colombiana
Aquichán
Archivos de Medicina
Avances en Enfermería
Biomédica
Biosalud
CES Medicina
Colombia Médica
Escuela Colombiana de Rehabilitación
Hacia la promoción de la salud
Iatreia
Infectio
Investigación y Educación en Enfermería
Medicina UPB
MedUNAB
Revista Ciencias de la Salud
Revista Colombiana de Anestesiología
Revista Colombiana de Cirugía
Revista Colombiana de Enfermería
Revista Colombiana de Nefrología
Revista Colombiana de Neumología
Revista Colombiana de Psicología
Revista Colombiana de Psiquiatría
Revista Colombiana de Radiología
Revista Colombiana de Cancerología
Revista Colombiana de Cardiología
Revista Colombiana de Ciencias Biológicas
Revista Colombiana de Gastroenterología
Revista Colombiana de Obstetricia y Ginecología
Revista Colombiana de Reumatología
Revista Cuidarte
Revista de Asma
Revista de Salud Pública
Revista Facultad de Medicina
Revista Facultad Nacional de Salud Pública
Revista Gerencia y Políticas de Salud
Revista Latinoamericana de Psicología
Revista Médica de Risaralda
Revista Médicas UIS
Revista Medicina
Revista UDCA Divulgación Científica
Salud UIS
Salud Uninorte
Universidad y Salud
Universitas Médica Javeriana

### Extracción de datos

Para el proceso de extracción de datos de los artículos seleccionados, se construyó una matriz en el programa Microsoft Excel™. De cada artículo seleccionado, se obtuvieron los siguientes datos: título, año, idioma, revista, autor principal (como autor principal se consideró al que aparecía de primero entre los autores), número total de autores, *software* de análisis cualitativo utilizado, tipo de enfoque cualitativo, número de participantes, tipo de muestreo, estrategias de recolección de información, cantidad de referencias, formación académica de pregrado del autor principal, formación de posgrado del autor principal, afiliación institucional del autor principal y tipo de institución (pública o privada).

### Procedimiento de análisis de información

Inicialmente, se hizo un listado de todas las revistas del área de salud consultando Publindex. Se priorizó dicha lista, identificando aquellas relacionadas con temas de salud pública, con lo que se seleccionaron 47 revistas. El proceso de extracción y análisis de datos se dividió en varias fases. Inicialmente, se recuperaron los artículos, y se almacenaron en un archivo con el nombre y el año respectivo. En una segunda fase, se depuraron los listados obtenidos revisando en varias ocasiones los títulos y el resumen para verificar que el artículo correspondiera al tema de interés. En una tercera fase, los artículos se revisaron en detalle, analizando las diferentes secciones, especialmente la sección de materiales y métodos, para obtener los datos de interés. Para apoyar el análisis de la información se utilizó el *software* estadístico SPSS 23™.

## Resultados

Del proceso de búsqueda de información entre el 2011 y el 2021, se eligieron 81 artículos originales que cumplieron los criterios de selección (cuadro 2). La revista en la que se publicó la mayor cantidad de artículos cualitativos enfocados en temas de salud pública, correspondió a la *Revista de Salud Pública*, con el 44,4 % (n=36) de las publicaciones incluidas en esta revisión, seguida por la revista *Avances en Enfermería*, con el 8,6 % (n=7) de los artículos (cuadro 3).

**Cuadro 2:** Listado de artículos incluidos

Acciones de familias de personas con discapacidad víctimas de desplazamiento forzado
Agencia social, sexualidad y embarazo en menores de 15 años
Alteraciones de patrones funcionales en personas con tuberculosis pulmonar, Villavicencio, Colombia
Ambientes escolares saludables
Análisis comparativo de las percepciones sobre el VIH/SIDA de varones homosexuales y bisexuales colombianos, con experiencia migratoria o sin la misma
Aspectos de la salud sexual y reproductiva de las mujeres adolescentes de tres comunidades del Resguardo Indígena
San Lorenzo, Caldas: trabajo doméstico, partería tradicional y procesos organizativos de mujeres indígenas
Atención domiciliaria y pandemia Covid-19: experiencia desde enfermería
Autobarreras de las mujeres al diagnóstico y tratamiento oportuno del cáncer de mama
Barreras de acceso a los servicios de salud: narrativas de mujeres con cáncer de mama en Colombia
Barreras para la eliminación de la malaria en Guapi-Cauca, Colombia
Centro de Desarrollo Humano Comunitario: programa para familias afrocolombianas desde la investigación acción participativa
Cómo entender la experiencia de personas que viven con VIH: implicaciones para la clínica y la investigación.
Comprensión de la gestión de la política pública de protección integral de la infancia en Chile
Conflicto armado en Colombia y misión médica: narrativas médicas como memorias de supervivencia
Conocimiento de las oportunidades perdidas de vacunación en profesionales no PAI (Programa Ampliado de Inmunizaciones) de Bogotá, D.C.
COVID-19 y sus imaginarios socioculturales en Latinoamérica: una herramienta para la salud pública
Cuidar al paciente con COVID-19: entre la incertidumbre y el deseo de salir adelante
Dinámicas e interacciones entre comunidad universitaria y salud pública 2.0
Discapacidad y trabajo. El otro entre discursos y hechos.
La drogadicción y su lugar en los procesos pedagógicos ¿un problema oculto o evidente?
El apoyo social: estrategia para afrontar el cáncer de cérvix
Efectos de la publicidad en el consumo de bebidas alcohólicas en escolares de Bogotá
El embarazo en adolescentes bogotanas: significado relacional en el sistema familiar
El plátano: indicador de hambre y escasez de alimentos en familias beneficiarias de programas alimentarios en Vigía del Fuerte, Colombia
Elderly and forced displacement in Colombia
Empoderamiento de líderes comunitarias afrocolombianas desde la Atención Primaria de Salud
Enseñanza de la salud pública en la formación del pregrado de enfermería en una universidad colombiana
Entre quimioterapias, herbolaria y espiritualidades. Estudio antropológico sobre el pluralismo terapéutico en adultos con cáncer en México
Estrategias para la eliminación de malaria: una perspectiva afro-colombiana
Estudio de caso: la gestión de la alimentación escolar en Santiago de Cali y Bogotá D.C.
Expectativa de enfermeiros brasileiros acerca do acolhimento realizado na atenção primária em saúde
Experiencia de implementación de un modelo de atención primaria
Experiencias de enfermeros de la atención primaria, partícipes del modelo de atención integral
Experiencias de vida en mujeres con cáncer de mama en quimioterapia
Experiencias y narrativas de mujeres con VIH, víctimas de violencia de pareja en Bogotá (Colombia)
Experimentando el rechazo y las decepciones del sistema de salud durante la experiencia del dolor crónico en el envejecimiento
Exposiciones rábicas en Colombia: evaluación del sistema de vigilancia desde los actores
Factores relacionados con las prácticas alimentarias de estudiantes de tres universidades de Bogotá
Formación de médicos y enfermeras para la detección temprana del cáncer de mama en México
Gender differences in the interpretation of experiences of patients with tuberculosis in Medellín, Colombia
Imaginarios de sexualidad en estudiantes universitario.
Jivi indigenous peoples: Family functioning and health care, an analysis from Community Health Nursing practices
El apoyo social: estrategia para afrontar el cáncer de cérvix
La actividad del salubrista: un análisis desde las clínicas del trabajo
La gestación en medio de la inseguridad alimentaria: percepciones de un grupo de adolescentes embarazadas
La lactancia materna desde la perspectiva de madres adolescentes de Bogotá
La salud pública en el continuo salud-enfermedad: un análisis desde la mirada profesional
Más allá de los síntomas: vivir con VIH es motor de cambio
Migración de venezolanos a Florida Central, Estados Unidos. Aspectos relacionados con la percepción de condiciones y necesidades de salud mental en 2019
Mujeres compañeras de migrantes: imagen social y búsqueda de servicios de salud sexual y reproductiva
Mujeres con cáncer de seno: experiencias y significados
Necesidades insatisfechas de cuidadores primarios de pacientes con cáncer de mama: percepción diádica
Percepción de violencia desde escolares de dos instituciones educativas de la localidad de Kennedy, Bogotá
Percepción de adolescentes sobre consumo de sustancias psicoactivas en entornos escolares. Estudio cualitativo.
Percepción de la comunidad universitaria sobre el consumo de sustancias psicoactivas en la Universidad de Antioquia, Medellín, Colombia
Percepción de las necesidades en salud mental de población migrante venezolana en 13 departamentos de Colombia. Reflexiones y desafíos.
Prevención de drogas. Buenas prácticas de trece programas de Colombia.
Percepções das agentes comunitárias de saúde sobre o cuidado prénatal
Promoção da saúde e atenção primária no cuidado às pessoas com doença crônica não transmissível
Relación médico-paciente: impacto en las campañas de promoción y prevención para personas con VIH, Medellín Repercussions of the COVID-19 pandemic from the childrens’ perspective
Representaciones sociales de universitarios sobre la abstinencia sexual y los condones como mecanismos de prevención
Representaciones sociales del consumo de drogas en un contexto universitario, Medellín, Colombia, 2000
Representaciones sociales del embarazo y la maternidad en adolescentes primigestantes y multigestantes en Bogotá
Representações de mulheres em idade fértil e profissionais de saúde sobre utilização de cuidados de saúde reproductiva
Retos para la prevención y el control del consumo de tabaco y sus derivados en mujeres en Antioquia, Colombia
Roles y desafíos de mujeres jefas de hogar con VIH/SIDA
Salud sexual y reproductiva de adolescentes en Chile: el rol de la educación sexual
Salud sexual y reproductiva de adolescentes: percepciones de los profesionales en enfermería
Significado de cuidado en las relaciones de personas que viven con VIH/SIDA
Significado de las vivencias de niños afectados por el VIH/SIDA, adscritos a un centro de atención y apoyo
Significados de la actividad física en la cotidianidad. Los lugares de la belleza y el placer en una práctica de salud
Situación de la enfermería en el desarrollo de la atención primaria en salud en Antioquia (Colombia): aproximación desde la perspectiva de los profesionales
The perceptions of adolescents concerning sexual and reproductive rights that favor the prevention of pregnancy at this stage.
Trayectos de vida familiar y estilos de vida: hipertensión arterial y diabetes mellitus II
Treatment adherence in people living with HIV: Rrelationship between an explanatory model, motives, and practices
Universidad y conducta suicida: respuestas y propuestas institucionales Bogotá 2004-2014
Vacuna contra el virus del papiloma humano en adolescentes: análisis mediante grupos focales
Violencia en el trabajo del sector público de la salud: una visión desde las personas trabajadoras. Bogotá, Colombia, 2011-2012
Vivencias y experiencias de individuos con ideación e intento suicida
Vivências de jovens em terapia antirretroviral para o HIV: estudo fenomenológico

**Cuadro 3:** Volumen de publicaciones de investigaciones cualitativas en salud pública según la revista, periodo 2011-2021

Revista	n	%
Revista de Salud Pública	36	44,4
Avances en Enfermería	7	8,6
Revista Facultad de Medicina	6	7,4
Revista Gerencia y Políticas en Salud	6	7,4
Revista Ciencias de la Salud	5	6,2
Revista Colombiana de Enfermería	4	4,9
Aquichán	3	3,7
Investigación y Educación en Enfermería	3	3,7
Revista Facultad Nacional de Salud Pública	3	3,7
Revista Colombiana de Psiquiatría	2	2,5
Biomédica	1	1,2
Colombia Médica	1	1,2
Revista Cuidarte	1	1,2
Revista Medicina	1	1,2
Revista MedUNAB	1	1,2
Universidad y Salud	1	1,2

**Figura 1:**
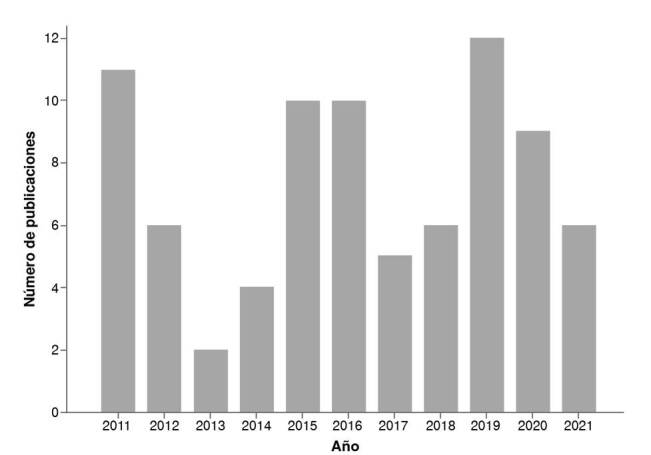
Investigaciones cualitativas según el año, 2011-2021

En cuanto al año de publicación, el 2019 se destaca como aquel con mayor número de artículos cualitativos en salud pública, con 12 artículos, seguido por el 2011, con 11 artículos. Desde el 2019, se evidencia una tendencia a la baja en el número de este tipo de publicaciones (figura 1).

Según el idioma de publicación, el 86 % (n=70) de los artículos se publicaron en español, el 7,4 % (n=6) se publicaron en portugués y, el 6 % (n=5), en inglés. En cuanto a los principales temas en los cuales se enfocaron los artículos, en el 12,3 % (n=10) de los artículos, el VIH/sida fue el tema predominante, seguido por el cáncer con el 11,1 % (n=9), la atención primaria en salud con el 7,4 % (n=6), y la salud sexual y reproductiva con el 7,4 % (n=6) (cuadro 4).

**Cuadro 4:** Tema principal de los artículos seleccionados, 2011-2021

	n	%
VIH/sida	10	12,3
Cáncer	9	11,1
Atención primaria en salud	6	7,4
Salud sexual y reproductiva	6	7,4
COVID-19	4	4,9
Alimentación	4	4,9
Drogadicción	3	3,7
Embarazo adolescente	3	3,7
Violencia	3	3,7
Discapacidad	2	2,5
Malaria	2	2,5
Migración	2	2,5
Políticas públicas	2	2,5
Suicidio	2	2,5
Sustancias psicoactivas	2	2,5
Tuberculosis	2	2,5
Vacunación	2	2,4
Actividad física	1	1,2
Agentes comunitarios	1	1,2
Consumo de alcohol	1	1,2
Consumo de tabaco	1	1,2
Vejez y desplazamiento forzado	1	1,2
Enfermedades no transmisibles	1	1,2
Enseñanza de la salud pública	1	1,2
Escuela saludable	1	1,2
Experiencia frente a servicios de salud	1	1,2
Exposición rábica	1	1,2
Lactancia materna	1	1,2
Promoción de la salud	1	1,2
Salubristas	1	1,2
Salud comunitaria	1	1,2
Salud mental	1	1,2
Salud pública 2.0	1	1,2
Sexualidad	1	1,2

Los autores que repiten como autores principales entre el 2011 y el 2021 en los artículos seleccionados, son M. M. Gómez y A. Knudson-Ospina, con dos menciones como autor principal cada uno, todos los demás autores cuentan con una mención como autor principal. Según el sexo del autor principal de los artículos, las mujeres fueron el autor principal en el 79 % (n=64) de los artículos y los hombres en el 21 % (n=17). En cuanto a la formación de pregrado del autor principal, en el 38,3 % (n=31) de los artículos no se detalló información al respecto, dado que se enfocan en la afiliación institucional. En el 19,8 % (n=16) de los artículos, la formación de pregrado del autor principal fue la enfermería y, en el 17,3 % (n=14), lo fue la medicina (cuadro 5).

**Cuadro 5:** Formación de pregrado del autor principal

	n	%
Sin dato	31	38,3
Enfermería	16	19,8
Medicina	14	17,3
Psicología	5	6,2
Antropología	4	4,9
Nutrición	3	3,7
Filosofía	2	2,5
Administración	1	1,2
Educación	1	1,2
Fisioterapia	1	1,2
Enfermera-Matrona*	1	1,2
Optometría	1	1,2
Trabajo social	1	1,2

*Pontificia Universidad Católica de Chile

**Figura 2:**
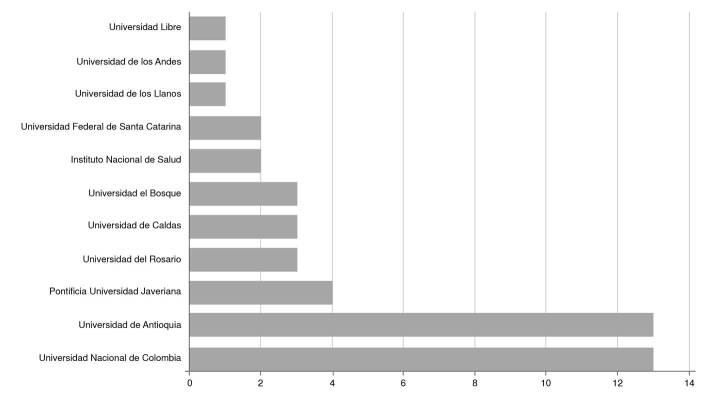
Afiliación institucional según autor principal

En relación con la afiliación institucional del autor principal, el listado lo encabezan la Universidad Nacional de Colombia y la Universidad de Antioquia, con 13 menciones cada una. El listado de las diez primeras menciones se detalla en la figura 2.

En relación con el país al que corresponde la institución referenciada en la afiliación institucional del autor principal, dado que existen iteraciones, se consolidó un total de 45 instituciones (una afiliación se reporta como *actividad independiente*). De las 45 instituciones, 21 (47 %) corresponden a instituciones privadas y 24 (53 %) corresponden a instituciones públicas. Ahora bien, al omitir la mención de una organización internacional con el fin de caracterizar el país de afiliación institucional, de las 44 instituciones restantes, el 63,6 % (n=28) de las instituciones corresponden a Colombia; el 11,3 % (n=5) a Brasil; el 7 % (n=3) a Chile; el 4,5 % (n=2) a México; y el 4,5 % (n=2) a España. Otros países, como Salvador, Portugal, Uruguay y Ecuador, cuentan con una mención cada uno (2,3 %, respectivamente).

**Figura 3:**
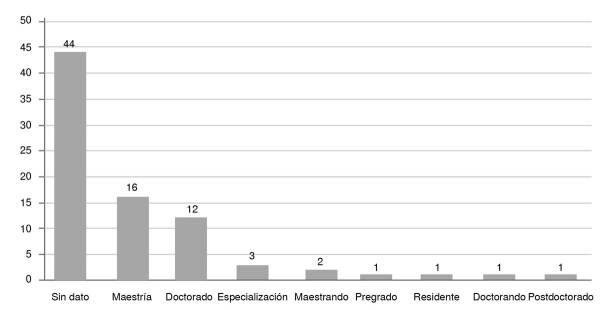
Nivel de formación académica máxima del autor principal

En cuanto a la mayor frecuencia del número de investigadores de los artículos, el 27,2 % (n=22) de los artículos estuvo integrado por tres investigadores, el 25,9 % (n=21) por dos investigadores, el 19,8 % (n=16) de los artículos por cuatro integrantes y el 12,3 % (n=10) de los artículos por un investigador. El promedio de investigadores por artículo corresponde a 3,3. Ahora bien, en relación con el nivel de formación académica máxima del autor principal, en 54,3 % (n=44) de las publicaciones se omite dicha información, en el 20 % (n=16) corresponde al nivel de maestría y en el 15 % (n=12) de los artículos corresponde a doctorado (figura 3).

En relación con el enfoque metodológico del estudio, el 17,3 % (n=14) de los artículos indicaron enmarcarse en la teoría fundamentada, el 16 % (n=13) señaló el enfoque fenomenológico y el 11,1 % (n=9) indicó el método etnográfico. Por otro lado, el 11,1 % (n=9) de los artículos no especificó un tipo de diseño cualitativo en particular (cuadro 6).

**Cuadro 6:** Diseño metodológico de los artículos cualitativos, 2011-2021

	n	%
Teoría fundamentada	14	17,3
Fenomenológico	13	16,0
Etnográfico	9	11,1
Sin especificar	9	11,1
Exploratorio	7	8,6
Interpretativo	7	8,6
Hermenéutico	5	6,2
Acción participación	3	3,7
Análisis de discurso	3	3,7
Descriptivo	3	3,7
Análisis de contenido	2	2,5
Estudio de caso	2	2,5
Análisis de crítica del límite	1	1,2
Antropología cognitiva	1	1,2
Construccionista social	1	1,2
Convergencia asistencial	1	1,2

Entre los estudios que recurrieron a la teoría fundamentada (n=14), en el 35,7 % (n=5) el tema principal se enfocó en VIH/sida, mientras, en el 28 % (n=4), el tema principal fue el cáncer. En cuanto a la cantidad de participantes, la menor cantidad reportada fue de cuatro y la mayor cantidad correspondió a 134. Por otro lado, de los artículos incluidos, el 75,3 % (n=61) no reportó el uso de *software* para el análisis cualitativo, el 21 % (n=17) mencionó Atlas Ti, el 1,2 % (n=1), Ethnograph, el 1,2 % (n=1), MaxQDA, y el 1,2 % (n=1), Nvivo.

En cuanto al tipo de muestreo, el 45,7 % (n=37) de los artículos no especificaron el tipo de muestreo realizado. Ahora bien, el 16 % (n=13) de los artículos indicó que el muestreo realizado fue por conveniencia y el 11,1 % (n=9) mencionó el muestreo en bola de nieve (cuadro 7).

**Cuadro 7:** Tipo de muestreo realizado

	n	%
No especifica	37	45,7
Muestreo por conveniencia	13	16,0
Bola de nieve	9	11,1
Muestreo teórico	8	9,9
Muestreo intencional	5	6,2
Saturación teórica	5	6,2
Muestreo de caso crítico	1	1,2
Muestreo intencional	1	1,2
Muestreo por conveniencia e intencional	1	1,2
No probabilístico-propositivo	1	1,2

En relación con las herramientas de recolección de información, el 82,7 % (n=67) de los artículos seleccionados recurrieron a entrevistas, el 32 % (n=26) utilizaron grupos focales, el 9,87 % (n=8), diarios de campo, el 14,8 % (n=8) utilizaron la observación participante, el 3,7 % (n=3), la revisión documental, el 1,2 % (n=1) utilizó dibujos, el 3,7 % (n=3), relatos de vida, y el 2,46 % (n=2), la cartografía social.

En cuanto a las referencias incluidas en los artículos seleccionados, la menor cantidad fue de 11 y la mayor cantidad fue de 70, y el promedio de las incluidas en los artículos fue de 28. El total de referencias en los artículos incluidos fue de 2.286, de las cuales 48,3 % (n=1.105) correspondieron a revistas científicas. Del total de estas referencias correspondientes a revistas científicas, el 29,6 % (n=328) eran de Latinoamérica; el 29,2 % (n=323) de Europa, el 22,2 % (n=246) de Estados Unidos; el 17 % (n=188) de Colombia; el 0,72 % (n=8) del África; el 0,72 % (n=8) de Asia, y el 0,36 % (n=4) de Oceanía.

## Discusión

En los últimos años, en Colombia, se ha incrementado la producción de investigación científica y la investigación cualitativa en salud no ha sido la excepción. En cuanto a temas de salud pública, la dinámica de la producción cualitativa ha sido variable desde el 2011 y el 2019 se destaca como el año con mayor cantidad de artículos cualitativos enfocados en salud pública. En el 2020 y en el 2021, dicha producción mermó, posiblemente por factores relacionados con la pandemia de COVID-19, como las inequidades en la financiación ^7^.

Ahora bien, dado que buena parte de las investigaciones cualitativas utilizan entrevistas o grupos focales, en una situación de pandemia, especialmente ante cuarentenas o confinamientos, esto puede afectar el desarrollo de investigaciones que requieren la interacción con los participantes, aunque, por otro lado, la pandemia de COVID-19 permitió la potenciación de herramientas tecnológicas, como los encuentros mediante videoconferencia. Por esto, gracias a los recursos tecnológicos, se llevaron a cabo investigaciones cualitativas que permitieron estudiar fenómenos relacionados con la pandemia de COVID-19 durante la misma ^8^.

En cuanto al idioma de publicación, cabe aclarar que no todas las revistas en el país publican solamente en español; algunas revistas como, por ejemplo, la *Revista Investigación y Educación en Enfermería* o la *Revista de Salud Pública,* publican artículos en portugués; también, está el caso de la *Revista Aquichán* que publica artículos en inglés. Esto puede representar un mayor alcance de la revista dentro de la comunidad científica y también brinda oportunidad para que autores de otros países publiquen en Colombia.

En el presente estudio, predominaron las mujeres como autor principal de los artículos, lo cual concuerda con otros estudios, como el de Carcausto-Calla y Morales-Quispe ^9^. En cuanto a esto, es importante considerar diferentes aspectos, ya que, en carreras como enfermería, medicina y psicología, el número de mujeres es mayor, aunque, en general, en carreras del área de la salud las mujeres representan la mayoría de los graduados, como lo evidencia el Observatorio Laboral para la Educación (OLE), el cual reporta que, en Colombia entre el 2001 y el 2020, el número de graduados en el área de la salud fue de 29.909 hombres frente a 51.459 mujeres ^10^. También, puede sugerir un creciente protagonismo de las mujeres dentro del mundo de la investigación, en este caso, en la investigación cualitativa enfocada en salud pública, y es claro que en las últimas décadas el número de investigadoras ha crecido y según Minciencias, para el 2019, Colombia contaba con 6.411 mujeres investigadoras que representan el 38 % de quienes se dedican a la investigación en el país ^11^.

En relación con los diseños de los estudios, el 11,1 % de los artículos incluidos en el presente estudio no especificaron el diseño metodológico utilizado y solo se menciona el estudio como *cualitativo*; dicho porcentaje es similar al reportado por Carcausto-Calla en el 2018, aunque dicho estudio se enfocó en temas de salud en general. Un elemento para destacar es el protagonismo de la teoría fundamentada como diseño principal de los artículos seleccionados.

Conocida como *grounded theory,* este tipo de investigación cualitativa fue desarrollada en 1967 por los sociólogos Barney Glaser y Anselm Strauss ^12^. La teoría fundamentada propone el método comparativo contrastante y se fundamenta en la ausencia de teoría preconcebida; parte de un área de estudio en particular y permite que la teoría emerja de los datos ^12^. Se refiere, entonces, a que la teoría que se construye se fundamenta en los datos, lo cual se realiza mediante un proceso inductivo para comprender la realidad de los participantes ^13^. En el área de la salud, la teoría fundamentada se ha utilizado de manera creciente para investigaciones sobre familia y adicciones.

En el presente estudio, en cuanto a temas de salud pública, la teoría fundamentada se utilizó para el estudio de experiencias sobre los servicios de salud, tuberculosis, sexualidad, uso de sustancias psicoactivas y, principalmente, cáncer y VIH/sida.

Cabe señalar que en el presente estudio se identificó la enfermería como principal productora de los estudios cualitativos en salud pública, seguida de cerca por la medicina. Claramente, la investigación cualitativa brinda grandes aportes en el estudio de aspectos subjetivos del proceso de salud-enfermedad como el autocuidado, la experiencia con la enfermedad, con las instituciones y con el personal de la salud. Notables aportaciones desde la enfermería se han realizado con diseños fenomenológicos, etnográficos y de investigación-acción, estos últimos, donde el investigador participa como agente de cambio ^14^. Con la investigación cualitativa, la enfermería, aunque también la medicina, cuentan con un gran aliado para profundizar en el estudio de diversos aspectos del campo de la salud pública, ya que el paradigma naturalista permite aproximarse a múltiples fenómenos relacionados con el cuidado de las personas, entender sus necesidades y optimizar el cuidado ^15^.

Un elemento importante producto de este estudio y que invita a la reflexión es la heterogeneidad metodológica y la presentación de los estudios. Aunque es claro que existen decenas de diseños de investigación cualitativa, se evidencian notables diferencias al momento del reporte. Por ejemplo, no todos los estudios que incluyen grupos focales detallan la cantidad de personas que participan y, en ocasiones, no es claro a cuánto corresponde la muestra. En otros casos, los estudios no detallan el tipo de muestreo realizado o bajo qué criterios se eligieron los participantes.

El uso de *softwares* que apoyen el análisis cualitativo es algo para destacar. Se puede mencionar que los estudios que apoyan el análisis con programas informáticos, como Ethnograph o Atlas Ti, omiten aspectos de su uso como la codificación o la inclusión de gráficos o redes semánticas; todo ello apunta a la necesidad de optimizar el rigor metodológico y la calidad del reporte de los estudios. Desde la década de los ochenta se han desarrollado programas informáticos para ello; en 1985, por ejemplo, apareció Ethnograph, el primer *software* para el análisis de datos cualitativos ^(16)^. Para 1989, apareció Atlas Ti, un *software* que vio la luz en la Universidad Tecnológica de Berlín en el contexto del proyecto Atlas y que haría su debut comercial en 1993 ^17^. Tanto Ethnograph como Atlas Ti corresponden a la tercera generación de estos programas, los cuales se conocen como de *elaboración teórica*^18^. Estos programas se han optimizado con el paso de los años y hoy se cuenta con más de 20 *softwares* de este tipo; sin embargo, pese a sus bondades, se usan con poca frecuencia, como lo evidencia la presente revisión, pues solo en el 24,6 % de los estudios se utilizó un *software* de análisis cualitativo.

Este estudio cuenta con algunas limitaciones, principalmente, en relación con que es posible que algún artículo no se haya incluido por no tratarse de una revisión sistemática. Sin embargo, se realizó una búsqueda exhaustiva y, por tanto, el estudio permite dar cuenta de la dinámica de la producción cualitativa en temas de salud pública en el periodo 2011-2021.

En cuanto a fortalezas, este estudio aporta a la comprensión de la dinámica de la investigación cualitativa en temas de salud pública en Colombia en la última década. También, es útil para dar cuenta de qué se ha hecho y qué falta por hacer, y en esta línea resalta muchas posibilidades para la salud pública en comunión con la investigación cualitativa.

De forma habitual, la investigación biomédica, especialmente la investigación clínica y epidemiológica, se ha enfocado en los pacientes; sin embargo, la investigación cualitativa permite abrir un espacio para dar voz a otros protagonistas del sistema de salud, a profesionales de diferentes áreas, como personal del área asistencial, personal de vigilancia en salud pública, personal administrativo y, por supuesto, a los pacientes y a sus familias; también, permite comprender, de mejor manera, múltiples fenómenos o problemáticas relacionadas con la salud pública. Además, se considera importante fomentar la investigación cualitativa en todos los niveles de formación del área biomédica en el país y en el momento actual de pospandemia, es relevante estimular la investigación cualitativa en los temas relacionados con la salud pública.
